# Hard Candy Production and Quality Parameters: A review

**DOI:** 10.12688/openreseurope.16792.1

**Published:** 2024-03-26

**Authors:** Baris Ozel, Sena Kuzu, Mehmet Ali Marangoz, Sarper Dogdu, Robert H. Morris, Mecit H. Oztop

**Affiliations:** 1 Department of Food Engineering, Middle East Technical University, Ankara, 06800, Turkey; 2 Durukan Confectionary, Ankara, 06935, Turkey; 3 Department of Physics and Mathematics, Nottingham Trent University, Nottingham, NG11 8NS, UK

**Keywords:** Hard candy, sucrose, glucose, storage, crystallization, characterization

## Abstract

Hard candies are sugar confections comprising mainly water and sucrose. Corn syrup, colorants and flavors are also usually added to hard candy formulations. The production of hard candy requires heating of the ingredients to very high temperatures to reduce moisture content and subsequent cooling to obtain a solid matrix. Cooling of the mixtures achieves the final, well known glassy state of the products. In this glassy state, the system is kinetically stable and molecular mobility is restricted, providing longer shelf life to hard candies. There are, however, several factors affecting the final quality and consumer acceptance of hard candies. Production methods and parameters, initial formulations as well as storage conditions all play a crucial role in the physicochemical, textural and sensory properties of hard candies. Addition of colorants and flavors also plays a vital role in the final quality. Although hard candy production is a simple process with few production stages, even small changes in the method of production and process parameters may induce substantial changes in the final product characteristics. Additionally, storage conditions such as temperature and humidity can change the product properties leading to graining and stickiness which are the two major problems for hard candies during storage. Both production and storage conditions should therefore be carefully chosen and controlled for desirable hard candy properties. This review addresses the general production methods and considers process parameters and quality parameters of hard candy products. Moreover, a comprehensive review of the related hard candy literature is also presented. The majority of hard candy reviews focus on specific methods and processes, but this review will present a more general frame on the subject.

## Introduction

Hard candies are a class of sugar confectionery produced by cooking a mixture of sucrose and corn syrup at high temperatures ( Sabbagh & Fagerson, 1979). The mixture of sugar mass is boiled up to maximum temperature and water content is reduced to minimum. The sugar mass, also known as a dough, is then cooled down to room temperature. The resulting products have a hard texture with glossy appearance ( Hartel *et al.,* 2011). Sucrose-free hard candies can also be produced by other substances such as sugar alcohols ( Hubbermann, 2016). Due to these processing conditions, hard candies or hard-boiled candies have low moisture content (2 – 3 %) at the glassy amorphous state ( Ergun *et al.,* 2010). The hard candy processing method is designed to obtain a product in glassy amorphous state below glass transition temperature (T _g_). In such glassy (vitreous) state, the system is kinetically stable and thus molecular mobility is severely restricted ( Sherwin & Labuza, 2006). This provides temporary stability to the hard candies during storage. However, changes in storage conditions such as temperature and relative humidity (RH) can quickly destabilize the products ( Roos & Karel, 1991). Sucrose recrystallization (graining), stickiness due to excessive moisture absorption or a combination of the two are two main problems encountered during storage of hard candies ( McFetridge *et al.,* 2004). In addition to processing and environmental conditions, hard candy composition also plays a crucial role in storage stability. Hard candies with high sucrose content may have a high T _g_ but they may be more prone to graining. On the other hand, hard candies with high corn syrup content may show some resistance to graining but they may suffer from excessive moisture uptake during storage which leads to an undesirable sticky texture ( Van Hook, 1961). Sugar-free ingredients may also lead to high levels of moisture uptake due to their hygroscopic character ( Raudonus *et al.,* 2000). A sticky texture is undesired due to the difficulties in unwrapping and reduced consumer acceptance. Therefore, it is crucial to control the hard candy composition, production process and storage conditions ( Hartel, 2002).

Although hard candy production is simple in comparison to many foods, even small changes in the composition and production parameters may severely influence the final product quality. The main quality parameters of hard candies include initial to final moisture content, temperature, pH, color, rheological and textural properties, incorporation, retention and release of flavor compounds, recrystallization and stickiness. Each of these parameters can exert distinct effects on final quality of hard candy products. For instance, temperature is an important parameter both in production and storage stages. As the temperature is increased during hard candy production, a more concentrated mixture with lower moisture content is obtained. However, extreme temperatures may induce some undesirable changes (e.g. sucrose inversion, caramelization.) in the hard candy matrix ( Hartel *et al.,* 2011). During storage, temperatures higher than the T _g_ of the hard candies promote a transition from the glassy to rubbery state where molecular mobility is increased. Under such conditions, hard candies may experience stickiness or graining depending on their compositions ( Balasubramanian *et al.,* 2016). Another parameter that impacts hard candy quality is the pH. The pH of the hard candy mixtures is generally adjusted by citric acid and provides the characteristic sour taste of hard candies ( Hubbermann, 2016). Visual properties are important and can be varied at the recipe stage. Opaque or transparent hard candies can be produced, for example transparent hard-boiled candies can be produced with sugar-free ingredients ( Lans *et al.,* 2018). Color pigments and flavours can be added to hard candy formulations to obtain different products ( Bund & Hartel, 2010). Since each flavor compound has distinct physicochemical properties, the resulting product quality is affected by the type and concentration of the added flavor compound. Retention of flavors and their release from hard candy matrix during consumption are also important factors influencing the final product quality ( Ergun *et al.,* 2010). Rheological or textural properties, on the other hand, contribute to the sensory properties and consumer acceptance of the hard candies. Since the number of literature studies about hard candies is limited, the main purpose of this review is to bring together all the hard candy studies and provide a general perspective on the matter. To this end, in this review materials that are generally used in hard candy formulations, hard candy production methods, process parameters and general quality and storage parameters of hard candies will be addressed. A summary of the selected hard candy studies is also presented in Table 1.

**Table 1.** Ingredients, process parameters and analyses of some hard candy studies.

Hard Candy	Ingredients	Cooking Temp.	Flavor/Phytochemical	Analyses	Reference
Caramel glass	Caramel, water	-	-	DSC, XRD, viscosity	( Cardoso & De Abreu, 2004)
Jagery candy	Jagery, gelatin, guar gum, xanthan gum, gum acacia, water	140 – 142 °C	-	Moisture content, organoleptic analysis	( Dinesh Kumar *et al.,* 2021)
Hard milk candy	Glucose syrup, milk, vegetable fats	118 – 130 °C	-	TPA ^1^, moisture content	( Figiel & Tajner-Czopek, 2006)
Black raspberry confection	Corn syrup, sucrose	150 °C	Black raspberry powder	TGA ^2^, DSC, rheology, texture, SEM, *in vitro* dissolution, sensory	( Gu *et al.,* 2015)
Sucrose-free hard candy	Isomalt, maltitol syrup, xylitol, water	170 °C	*Cudrania tricuspidata* fruit extract	Moisture content, soluble solid content, color, hardness, sensory	( Jeon *et al.,* 2021)
Capsaicin candy	Xylitol	120 °C	Capsaicin	DSC, XRD, hardness, *in vitro* release, immunohistochemistry	( Jiang *et al.,* 2019)
Hand-made hard candy	Calcium maltobionate, reduced isomaltulose	-	-	DSC, TRA ^3^, color, water content	( Kawai *et al.,* 2019)
Commercial hard- boiled candy	Glucose syrup, granulated sugar, citric acid, water	145 °C	Anthocyanin	Color analysis (colorimetry, spectrophotometry, LPAS ^4^	( Kovács *et al.,* 2017)
GOS ^5^ glassy confection	Sucrose, corn syrup, GOS, water	153 °C	-	DSC, TGA, moisture content, texture, polarized light microscopy, sensory	( Lans *et al.,* 2018)
Sugar glass	Sucrose/lactose, corn syrup/whey, water	-	-	NIR microspectroscopy (moisture penetration), moisture content	( Liang *et al.,* 2007)
Confectionery with carrot powder	Sucrose, corn syrup, water	110 °C	β-carotene	Retention and storage stability of β-carotene in hard candies	( Shaaruddin *et al.,* 2019)

^1^Texture profile analysis, ^2^Thermogravimetric analysis, ^3^Thermo rheological analysis, ^4^Laser-based photoacoustic spectroscopy, ^5^Galacto-oligosaccharide

## Hard candy production

### Materials

The main ingredients of hard candies are water, sucrose and, in many formulations, corn syrup. Corn syrup is added to formulations as a doctoring agent to inhibit sucrose crystallization during production and storage ( Lans *et al.,* 2018). Corn syrup contains glucose, fructose and higher molecular weight (MW) oligosaccharides. There are different mechanisms by which corn syrup sugars inhibit sucrose crystallization which will be discussed in the following sections.

Besides simple sugars, sugar alcohols such as xylitol ( Jiang *et al.,* 2019), isomalt ( Raudonus *et al.,* 2000) and maltitol ( Jeon *et al.,* 2021) have been used alone or in combination to produce hard candies. Jiang *et al.* (2019) have recently used xylitol alone to produce capsaicin containing sucrose-free hard candies whereas Jeon *et al.* (2021) combined isomalt, maltitol syrup and xylitol. Isomalt was also reported to be used in combination with hydrogenated starch hydrolysates, polydextrose and some other polymeric additives ( Raudonus *et al.,* 2000). Honey ( Sahlan *et al.,* 2019), gelatin ( Saint-Eve *et al.,* 2011) and galactooligosaccharide ( Lans *et al.,* 2018) based hard candies have also been produced. The use of calcium maltobionate with sugar and calcium maltobionate with reduced isomaltulose for hard candy formulations have also reported ( Kawai *et al.,* 2019). Traditional ingredients specific to certain regions such as jaggery on the Indian subcontinent have also been utilized for hard candy production ( Dinesh Kumar *et al.,* 2021). In the same work, jaggery was combined with hydrocolloids including xanthan gum, guar gum and gum acacia to improve the candy texture. Hard candies with soft fillings and a hard outer coating are also often produced and consumed. For instance, a caramel coating for fillings imparted desirable sensory and textural properties to hard candies ( Figiel & Tajner-Czopek, 2006).

Other essential materials for hard candies are organic acids and flavors. Generally, citric acid (0.1 – 1.0 %, w/w) is included in hard candy formulations for pH regulation and product preservation ( Bund & Hartel, 2010). Citric acid is abundantly used by the food industry as an acidifier due to its fruity taste and high availability ( Marques *et al.,* 2020). However, malic acid is also used for such purposes with its smooth taste and preservative properties. DL-malic acid with a low melting point shows promise as an alternative to citric acid in hard candies ( Kwon *et al.,* 2019). Malic acid was also found to provide a longer flavor retention in the mouth than citric acid, at low concentrations ( Lugaz *et al.,* 2005). A variety of flavors can be incorporated into hard candy formulations. Some examples include L-menthol (mint flavor), vanillin (vanilla flavor), 1,8 cineole (eucalyptus flavor), citral (lemon flavor), propylene glycol and benzaldehyde (cherry flavor) ( De Roos, 2003; Reineccius *et al.,* 2004; Schober & Peterson, 2004; Spanemberg *et al.,* 2019). Complexation of flavors with other substances to increase flavor retention has also been employed. Reineccius *et al.* (2004) complexed L-menthol with β-cyclodextrin to flavor their hard candies and a substantial increase in L-menthol retention was achieved.

### Production method and process parameters

Production of hard candy includes two main processes: heating the candy mixture to high temperatures and a subsequent cooling process. There are however many details that must be considered for a desirable final product such as initial moisture content of the mixture, composition of the hard candy (e.g. sugars, syrups, additives) final temperature achieved during heating, addition of acid and flavors (concentration and timing of the addition) and rate of cooling. In addition, packaging and storage conditions should also carefully be controlled. Otherwise, hard candies with undesirable taste, texture and appearance could be obtained ( Ergun *et al.,* 2010).

Heating is applied to hard candies to decrease the water content of the mixture since excess water has a plasticizing effect on hard candy texture ( Roos & Karel, 1991). A final moisture content below 5 % (w/w) should be achieved for hard candies ( Bussiere & Serpelloni, 1985). A candy mass is first prepared by mixing sugar (sucrose), water and corn syrup (glucose syrup). This mass is homogeneously mixed and then heated up to 135 – 160 °C with no stirring. Organic acid, flavors, color pigments and other additives are added to the mixture before cooling. down to room temperature ( Hartel *et al.,* 2011). This is the general production process for hard candies but the parameters change for different formulations and applications. There are many studies in the literature that reported various process parameters for hard candy production. If the hard candy mass includes only sucrose and water, the mixture is generally boiled up to the standard hard-crack temperature of hard candies which is between 145 °C and 160 °C, depending on the ratios of the sugar and water ( Jiang *et al.,* 2019; Swer *et al.,* 2019). On the other hand, different maximum heating temperatures for different formulations were reported such as 120 °C for xylitol based hard candies ( Jiang *et al.,* 2019) and 160 °C for isomalt candies ( Raudonus *et al.,* 2000). Production of caramel hard candies required the heating of the mixture of glucose syrup, milk and vegetable fat between 118 and 130 °C ( Figiel & Tajner-Czopek, 2006; Minifie, 1989). Although many producers simply prefer cooking of the hard candy mass at atmospheric pressure, vacuum may also applied during the final stages of cooking process especially in commercial hard candy production. The application of vacuum reduces the final temperature requirement providing less sucrose inversion and aids moisture removal achieving similar results in a shorter duration. Thermal degradation of added pigments is also reduced under vacuum conditions ( Reinheimer *et al.,* 2010). For instance, Sabbagh and Fagerson (1979) have heated their hard candy mixture up to 135 °C at atmospheric pressure and then applied vacuum for five minutes at 165 mmHg. In this way, they obtained hard candies with final moisture content of 2.14 % (w/w) after cooling.

Another critical step in hard candy production is the addition of organic acid and other additives during the heating and cooling process. Citric acid is generally added to the mixture at the end of heating and just before cooling in order to reduce its sucrose inversion effect at elevated temperatures ( Nadaletti *et al.,* 2011). Flavor and color pigment addition is also employed after heating the mixture to minimise undesirable changes. Heat stable pigments should however still be chosen for hard-boiled candies since the temperature at which they are added is still high. Generally, heat stability of added colors up to 145 °C is required ( Hubbermann, 2016). Under some conditions, coloring agents could be added to the candy mass at the later stages of cooling but this should be carefully controlled since the candy mixture is highly viscous. If color addition is undertaken at temperatures where the viscosity of the mixture prevents homogeneous mixing, the resulting product would lose its consumer acceptance. Nonetheless, color pigment addition between 110 and 140 °C was demonstrated to be possible by several studies allowing the use of pigments with less desirable high temperature stability ( Hubbermann, 2016; Sharma & Ghoshal, 2021). Despite the advantage of this approach, the use of heat stable flavors and color pigments is still the best alternative. For instance, anthocyanins and carmine are heat stable but red beet pigments are not. Since the pH of hard candies is around 3.0 – 4.0, the pigment to be used should also be acid stable. Thus, spirulina cannot be simply used for hard candy coloring purposes since it is acid labile and precipitates at low pH conditions. The added color should also be in concentrated form otherwise excessive water is introduced to the candy mixture which would reduce the final quality of the product ( Hubbermann, 2016).

Similar to colorants, addition of flavours to hard candies is also a challenging process. Producers usually add flavors at the final stage of heating to minimise possible damage. Spanemberg *et al*. (2019) cooked their syrup at 145 °C under vacuum and added lemon flavor just after cooking. In another study, green tea extract and *amla* powder were added to the hard candy mixture at the cooking temperature (150 °C) but at the possible latest stage of heating in order to achieve maximum natural vitamin retention ( Veeranna *et al.,* 2021). Heat sensitive materials should however be added at lower temperatures. Jeon *et al.*(2021) added *Cudrania tricuspidata* fruit extract at around 112 – 115 °C that was much lower than the cooking temperature at 160 °C. It should also be noted that the low moisture character of hard candies enables the addition of a great variety of substances as additives including freeze-dried materials. It was previously reported that freeze-dried raspberry powder was successfully incorporated into the sucrose – corn syrup hard candy mixture ( Gu *et al.,* 2015).

The final stage of hard candy production is the cooling process. In some cases, cooked hard candy mass is placed in molds and left to cool at room temperature ( Jeon *et al.,* 2021). However, in most cases, candy mass is subjected to initial cooling to 70 – 85 °C and shaped at that temperature prior to final cooling to 20 – 30 °C. Raudonus *et al.* (2000), firstly cooled isomalt hard candies down to 70 – 80 °C on a cooling table at 60 – 70 °C and then formed candies with a motor-driven drop roller. In another study, the candy mixture was tempered in a stainless-steel band with 50 °C water and then stamped and formed. Finally, the formed candies were cooled to 30 °C ( Spanemberg *et al.,* 2019). In industry, cooling is also performed by tunnel cooling where cold air is applied to the hard candies and process parameters such as cooling time, velocity and temperature of the cooling air and candy size determine the final quality of the product ( Reinheimer *et al.,* 2013). In this approach, the cooked hard candy dough is first tempered and mixed for homogeneous temperature distribution. The dough is then formed into a roll which is cut and stamped to complete the candy forming process. Finally, the formed hard candies are cooled in a cooling tunnel to room temperature ( Reinheimer *et al.,* 2010).

## Quality and storage parameters

### Moisture content

Hard-boiled candies have a low moisture content range of 2 – 3% ( Sharma & Ghoshal, 2021). This low moisture content produces a low water activity providing microbial, physical and flavor stability to hard candy products. If the water content in hard candies reaches unacceptably high levels (above 5%), water acts as plasticizer by reducing the T _g_ and disturbs the glassy structure ( Raudonus *et al.,* 2000) leading to sticky products with unacceptable texture. Presence of excess water is also the main cause of sucrose graining ( Bund & Hartel, 2010). Thus, a low moisture content at the desired levels is essential for longer shelf-life. Increasing moisture levels also affects the physicochemical and textural properties of the hard candy products. For instance, increase in moisture level from 1.3 to 2.0% decreased the cutting force, hardness and cohesiveness of hard honey candies ( Sahlan *et al.,* 2019).

Initial and final moisture contents of hard candies can be determined by Karl Fischer method ( Spanemberg *et al.,* 2019). Some researchers determine the initial moisture content of their hard candies by Karl Fischer method and then use gravimetric method to find absorbed water level ( Pushpadass *et al.,* 2014). Oven methods and halogen moisture analyzers can also be used for moisture content determination ( Jeon *et al.,* 2021). The initial moisture content of hard candy formulations should be carefully monitored since excessive moisture levels in the formulation may retard or even prevent the hardening of the mixture during processing ( Shukla & Kandra, 2015). Presence and type of added materials ( *e.g.* hydrocolloids, gums) also impact the final moisture content of hard candies. Dinesh Kumar *et al.* (2021) reported that increasing the gum concentration (xanthan gum, guar gum, gum acacia) in formulations resulted in a rise in the moisture content of the hard candies ( Dinesh Kumar *et al.,* 2021). Xylitol-based hard candies also experienced a higher final moisture content after production ( Jeon *et al.,* 2021). Flavor retention is another parameter that is closely related to the moisture content. The low moisture content of hard candy products protects flavors by retarding their release, thereby minimizing their loss during high-heat treatment ( Reineccius *et al.,* 2004).

### Temperature

Temperature is one of the most important factors affecting hard candy quality both during production and storage. As previously discussed, high temperatures (135 – 160 °C) are required for hard-boiled candy production ( Hartel *et al.,* 2011). Temperature also affects the cooling regime of the boiled hard candy mixtures. Cooling at room temperature as well as cooling in temperature-controlled environments (cooling tunnels *etc*.) can be applied to products. Shaping of the mixtures can also be performed at different temperatures depending on composition of the formulations ( Reinheimer *et al.,* 2013). Cooling of hard candies in cooling tunnels aims to reduce the range of temperature distribution within the individual products. Otherwise, a non-uniform temperature distribution may be observed which may lead to quality problems such as fragility, deformation and aggregation ( Reinheimer *et al.,* 2012). Optimization of the hard candy cooling process is however not an easy task. There is a necessary trade-off between economy and quality aspects. The optimum cooling conditions that would result in minimum temperature difference between the center and the surface of a hard candy sample require lower cooling-air velocities and higher cooling-air temperatures. Nevertheless, such conditions increase the residence time of hard candies in the cooling unit which negatively affects the process economics ( Reinheimer *et al.,* 2013). Since we have previously discussed the effects of temperature during the production step in the section titled ‘ ****Production method and process parameters****’ herein, we are going to focus on effects of temperature mainly during storage of the products.

**Figure 1.**
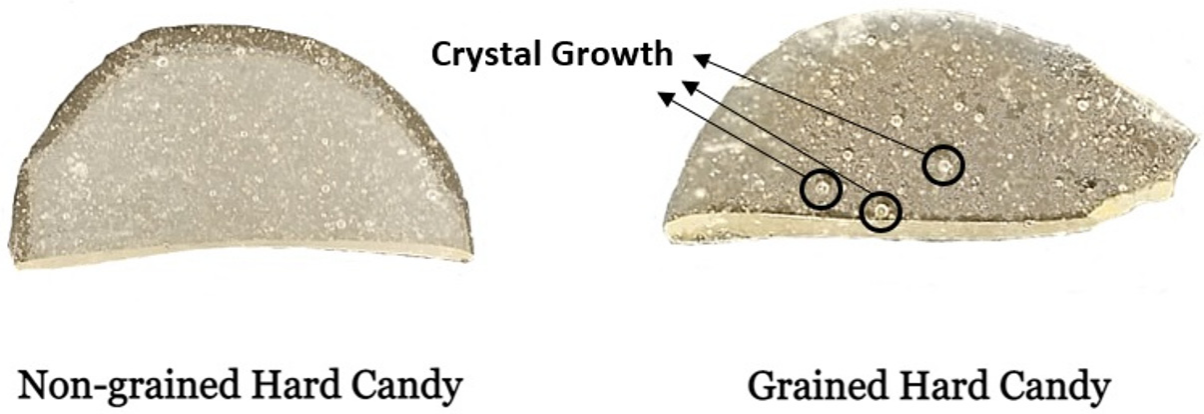
Photographic images showing graining in hard candies. Examples of areas with enhanced crystal growth are labelled in the right hand image of a grained hard candy.

***Glass transition temperature.*** Glass transition temperature (T _g_) has a pronounced effect on product quality and stability. Storage of hard candies at temperatures higher than their T _g_ has detrimental effects on quality attributes ( Tan & Kerr, 2017). Storage temperatures below T _g_ maintain the glassy state of the product where molecular mobility is restricted. When temperature is increased above T _g_, molecular mobility increases and the product loses its vitreous state ( Balasubramanian *et al.,* 2016). Thus, various physicochemical changes within the products can take place. For instance, sucrose crystallization during storage becomes possible after temperature exceeds T _g_ of the product. Increased molecular mobility firstly induces sucrose crystal nucleation and then subsequent crystal growth as shown in Figure 1. Temperature-induced crystallization is usually observed in the form of internal graining. In this form of graining, growth of previously formed crystals during hard candy production begins in the glassy matrix ( Bund & Hartel, 2010). The undesired results of this process include textural and sensorial deterioration and loss of flavor ( Hartel, 2002). Variations in the temperature during transportation and storage can also affect the crystallization behavior of hard candies. Changes in crystal number, size, shape and orientation could be observed when temperature is not strictly controlled during packaging, transportation and storage ( Lifran *et al.,* 2007).

**Figure 2.**
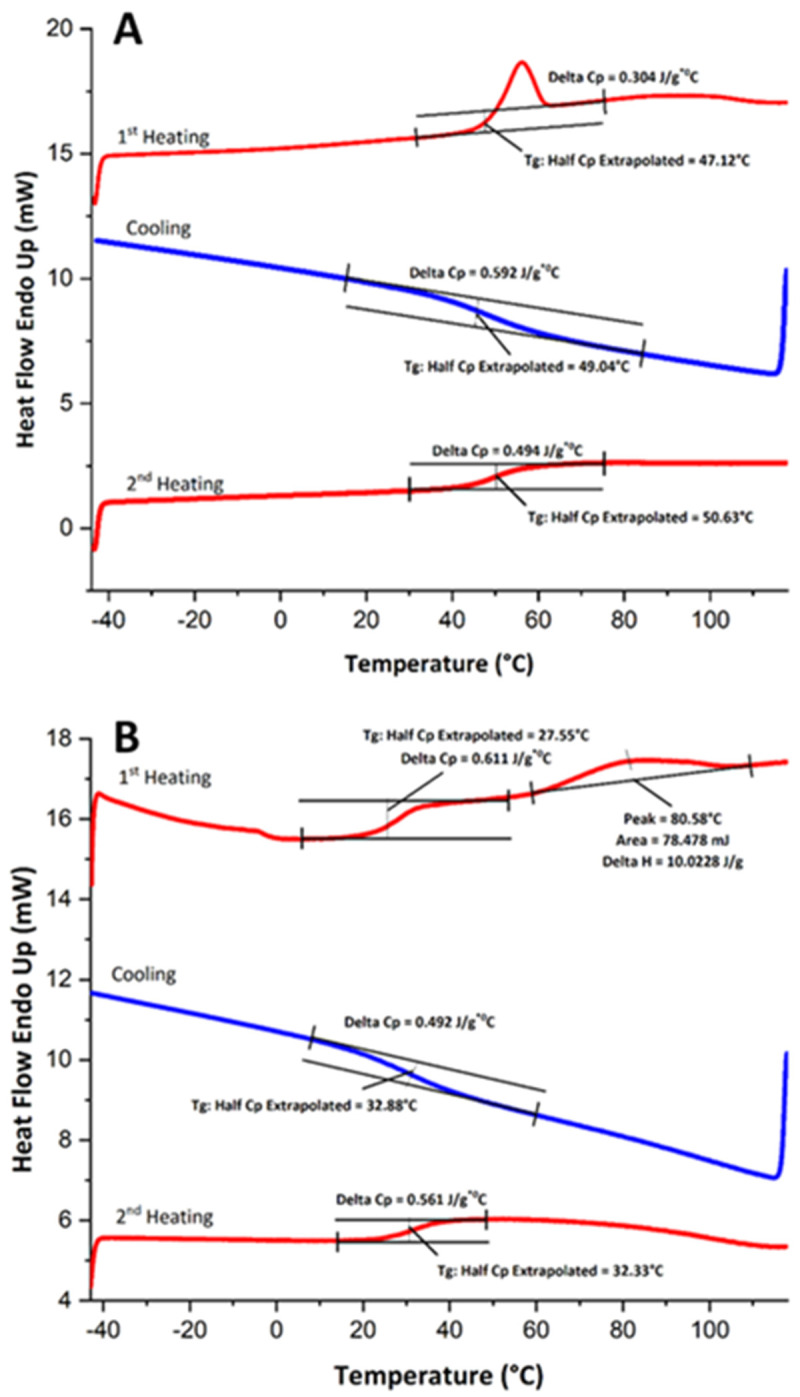
DSC curves of the hard candies with high crystallinity ( **A**), and low crystallinity ( **B**) fractions.

Composition of hard candies is another factor which affects T _g_. For instance, water acts as a plasticizer and reduces T _g_ at high levels ( Roos & Karel, 1990). Furthermore, hard candies are complex mixtures with many ingredients which may result in a glass transition range rather than a single glass transition temperature ( Saavedra-Leos *et al.,* 2012). Therefore, determination of an exact T _g_ may not be possible. Generally, differential scanning calorimetry (DSC) and thermal rheological analysis (TRA) are used for determination of T _g_ ( Kawai *et al.,* 2019). DSC is a classical thermal analysis based on characterization of the changes (heat flow or temperature) between the analyzed sample and a reference pan. The DSC technique requires a small sample size and provides high sensitivity. However, the formed DSC peaks are highly dependent on the rate of heating and cooling which would induce some inconsistency problems ( Bund & Hartel, 2010). Figure 2 shows the real-time DSC analysis of two hard candy samples with different crystallinity values. Most recently, the use of TRA was introduced to detect T _g_ of hard candy systems as a thermomechanical approach ( Kawai *et al.,* 2019). In this method, T _g_ is detected as force-drop of baseline in force vs temperature curves. Mechanical T _g_ is the temperature at which the onset point of force-drop is observed ( Boonyai *et al.,* 2007). However, mechanical T _g_ does not always correspond to the calorimetric T _g_ thus mechanical T _g_ should be calibrated to calorimetric T _g_. Preliminary experiments, where varying compositions (water and other ingredients) are applied, should be conducted for such a calibration ( Kawai *et al.,* 2019). Hard candy samples can be placed directly in the equipment for T _g_ analysis by TRA ( Sogabe *et al.,* 2018).

### pH

Hard candies have a pH range of three – four due to the presence of food acids in their formulation. Generally citric acid is used to provide the characteristic sour taste to the hard candies. Malic acid is also used to a lesser extent ( Lugaz *et al.,* 2005). pH is effective on many hard candy quality parameters such as color, taste, sucrose inversion and pigment stability ( Hubbermann, 2016). pH of the hard candy mixture influences the color shade of the product. Since some color pigments are susceptible to acidic pH values, such pigments may show a variation in their color in hard candy formulations. Acid-stable color pigments are therefore preferred in candy mixtures. The same situation is also valid for other pigments. Anthocyanins, for instance, could be used in such formulations due to their resistance to low pH. Many hard candies contain fruit flavors that are compatible with acidic pH. However, pH of these systems should also be controlled since a small change in pH may demonstrate distinct changes in appearance, flavor and color. Addition of ingredients to the candy matrix other than sugar was reported to induce large variations in pH. Saint-Eve *et al.* (2011) reported an increase in pH from 2.4 to 4.0 when they added 15 % (w/w) gelatin into their hard candy formulations. The resulting hard candies also showed variations in perceived sourness due to the variations in pH ( Saint-Eve *et al.,* 2011). pH can also drive the sucrose inversion process that occurs during the heating step of hard candy production. Generally, low pH accelerates sucrose inversion in combination with high temperature and high RH. Inversion of sucrose to glucose and fructose alters the physicochemical properties of the samples in terms of storage stability, texture, taste and appearance. Addition of lactate solution to hard candy formulations can also alter pH. Thus, lactate addition could alternatively be used for the pH regulation of the hard candy mixtures ( Nadaletti *et al.,* 2011).

### Color

Color is another important quality parameter of hard candies. Coloring agents could be added to the formulations during production. However, in some formulations, no colorant is used. If color pigments are added to hard candies, these pigments should be resistant to acidic and high temperature conditions. On the other hand, the low moisture content of hard candy products is an advantage since most natural color pigments are stable at such low water activity levels ( Sharma & Ghoshal, 2021). The amorphous nature of hard candies aids uniform dispersion of color within the product. Graining of hard candies during storage results in color loss since growing crystals exclude color pigments. An undesired mottled appearance may be observed if heterogeneous graining occurs during storage ( Bund & Hartel, 2010). Generally, water-soluble color pigments are added to hard candy mixtures since water-soluble pigments provide a transparent appearance. This can also be achieved in sugar-free hard candies ( Hubbermann, 2016). In contrast, pigments with lipophilic nature may impart a cloudy – opaque appearance due to emulsification.

We have focused on the color stability during production in section “production method and process parameters”, but stability of color during storage is also a critical parameter. Color stability during storage is a shelf-life limiting factor which is closely related with the storage and packaging conditions. Firstly, light exposure can be a problem, thus transparent packaging should be avoided. Moreover, oxidation of the color pigments is also possible since the candy packaging materials are mostly gas permeable (plastic, paper etc.). Coloring agents are mostly present at the surface of the hard candy material and these pigments may undergo degradation if the packaging material and the storage conditions are not carefully chosen and maintained. Effects of ingredients on color should also be considered. For instance, presence of polyols revealed a better color acceptance in sucrose-free hard candies. Transparency of such candies was reported to increase since interactions between the maltitol syrup components and xylitol affected the solubility of the colorant ( Jeon *et al.,* 2021). However, in another study, addition of hydrocolloids and gelatin did not affect the color of the final hard-boiled candy which the authors concluded was since the quantities of the added materials were not sufficient to induce significant changes in color ( Dinesh Kumar *et al.,* 2021).

Colorimetry is widely used for color detection of hard candy materials. The colorimetric method provides a quantitative measurement of color concentration of a substance by measuring its absorbance of a specific wavelength of light. Spectrophotometry is also used for the same purposes. Additionally, laser-based photoacoustic spectroscopy (LPAS) has been introduced as a fast and direct method for determination of color agents. Kovacs *et al.* (2019) showed that LPAS could be used for quick determination of anthocyanin and β-carotene in hard candies at 532 and 473 nm, respectively. Since the results obtained by colorimeter are affected by the physical condition of the samples, LPAS could be considered as an alternative providing less time consuming analysis and minimum sample preparation ( Kovács *et al.,* 2019). LPAS was also reported to eradicate the dissolution problem of spectrophotometric analysis where samples must be dissolved before analysis. In this way, the effect of pH on the results is eliminated. In LPAS, the sample to be analyzed is irradiated by radiation beam. A fraction of the absorbed energy is then converted to heat. Thus, the temperature of the sample starts to oscillate at the same frequency of the modulated radiation. The generated thermal waves reach the surface of the sample and induce periodic heating and cooling of the surrounding gas contacting layer. Due to this periodic heating and cooling, the surrounding gas layer expands and contracts and produce acoustic waves. These waves are detected by a sensitive microphone as a voltage. This signal is susceptible to the changes in the analyzed colorant concentration with higher anthocyanin contents, for instance, producing stronger photoacoustic signals ( Kovács *et al.,* 2017).

### Rheological and textural properties

Rheological analysis of hard candy mixtures can provide some information on the physicochemical properties of the samples. Raudonus *et al.* (2000) stated that addition of oligomeric or polymeric structures (hydrogenated starch hydrolysate or hydrogenated polydextrose) increased the viscosity of isomalt melts. Rheological properties of hard candy dough also affect the final textural attributes, thereby the dynamics of aroma and taste perceptions ( Saint-Eve *et al.,* 2011). Thermo rheological analysis (TRA) of hard candy mixtures including thermal mechanical analysis ( Chang & Randall, 1992), thermal mechanical compression testing ( Boonyai *et al.,* 2007) and phase transition analysis ( Avaltroni *et al.,* 2004), is another approach to determine the thermo-rheological properties of the samples. Generally, a texture analyzer is attached with a heating stage and temperature controller for TRA measurements.

The texture of hard candies is another crucial quality parameter. Hard candies are amorphous glassy systems that can experience changes in the desired texture and sensory properties. Such changes are usually induced by crystallization, stickiness problems, or both during production and storage leading to quality deterioration. Crystal formation and growth changes the crystal size distribution in hard candies which would eventually result in undesired texture (grainy texture) and poor sensory characteristics (loss of flavor, unacceptable appearance) ( Bund & Hartel, 2010). The best hard candy products are associated with hard texture and a clear, glossy appearance. Sticky hard candies with pale color are usually not accepted by consumers. However, an excessively hard candy may induce a decrease in perceived taste and aroma intensities ( Boland *et al.,* 2006). Variations in the hardness of a hard candy is also capable of changing the aroma-taste sequences perceived during consumption. For instance, hard candies with high gelatin concentration (high firmness) were described to initially have a butter sensation then strawberry perception. On the other hand, softer candies (low firmness) with much lower gelatin concentration were described to initially have a sour perception followed again by a strawberry perception ( Saint-Eve *et al.,* 2011). Here, the textural properties of the hard candy samples influenced the residence time of candy in the mouth, oral behavior and stimuli release. Higher gelatin content increased the firmness of the hard candy and consequently influenced the melting time of the candy in the mouth. This changed the time spent in mouth (residence time) before swallowing and thus the perception of the product by consumers ( Choi & Regenstein, 2000).

Hard candy content, especially water proportion, affects the textural properties. Distribution of water within the samples is also important in terms of textural attributes. Increasing water content of hard candies are generally associated with lower hardness ( Sahlan *et al.,* 2019). If the resulting softer texture combines with a sticky surface, the overall acceptability of these products decreases significantly ( Ergun *et al.,* 2010). Substitution of sucrose with doctoring agents and other substances such as sugar alcohols has also great impacts on textural properties of the resulting product ( Roos & Karel, 1993). Xylitol was previously reported to decrease the texture acceptability of hard candies especially at high levels ( Jeon *et al.,* 2021). Substitution of refined sugar with jaggery also produced an undesirable chewy texture. However, addition of 0.75 % (w/w) gelatin to jaggery based hard candies exerted much better texture and appearance ( Dinesh Kumar *et al.,* 2021). Sugar inversion during the heating process of hard candy production should also be controlled since it also has substantial effects on final product texture. In-line, real-time optical rotation measurement systems could be employed to allow instant adjustments of temperature, processing time and acid addition during production. In this way, monitoring and controlling of sugar inversion could be improved ( Huerta-Ruelas *et al.,* 2008). Hard candy texture can be analyzed in detail by texture profile analysis (TPA). However, simple instrumental tests such as simple strength tests (cutting tests) could also be applied for more rapid measurements ( Figiel & Tajner-Czopek, 2006).

### Flavor incorporation, retention and release

Many flavor compounds are incorporated into hard candy formulations. Prolonged retention of such compounds in the hard candies and their gradual release during consumption are the main desired characteristics. There are many studies which applied different aroma and flavor compounds to different hard candy formulations. Jiang *et al.* (2019) for example incorporated capsaicin to xylitol-based hard candies and investigated the retention time of these candies in mouth as well as the long-term release of capsaicin. They stated that the high hardness of the samples prolonged the retention time of candies in mouth and provided a slow release ( Jiang *et al.,* 2019). Swer *et al*. (2019) prepared traditional sucrose based hard candies and incorporated freeze-dried anthocyanin pigments obtained from Sohiong ( *Prunus nepalensis L.*) into their formulations. During a storage period of 90 days at room temperature, hard candies did not show a significant decrease in total anthocyanin content ( Swer *et al.,* 2019). Gu *et al.* (2015) added freeze-dried black raspberry powder into hard candies prepared with water, corn syrup and sucrose for controlled release purposes. The raspberry powder content reached 22 % in hard candies with 59 % of original anthocyanins retained after processing. However, hard candies demonstrated a fast release of the encapsulated material. The final release time was found as 90 min in artificial saliva dissolution studies ( Gu *et al.,* 2015). This fast release character of hard candies could be attributed to their smooth surface microstructure. Gu *et al.* (2015) investigated the surface microstructure of the candies with scanning electron microscope (SEM) and reported a smooth surface with freeze-dried raspberry particulates dissolved in it. Furthermore, hard candies are composed of nonpolymeric materials, thus, unlike polymeric materials, they can dissolve instantaneously with their dissolution controlled by external mass transfer resistance *via* a liquid layer that is adjacent to the solid-liquid interface ( Miller-Chou & Koenig, 2003). Shaaruddin *et al.* (2019) investigated the β-carotene stability in traditionally produced hard candies. In this study, different wall materials such as maltodextrin, resistant maltodextrin, octenyl succinate anhydride starches and HICAP-100 (a modified food starch derived from waxy maize) were used to produce carrot powders and the efficiency of these wall materials in β-carotene retention in hard candies was evaluated. Carrot powders produced with HICAP-100 showed the highest β-carotene retention after the hard candy production process whereas carrot powders produced with octenyl succinate anhydride starch wall material exhibited longer β-carotene stability during storage with the lowest degradation rate constant ( Shaaruddin *et al.,* 2019). Reineccius *et al.* (2004) stated that complexation of L-menthol (mint flavor) with β-cyclodextrin dramatically increased the retention of L-menthol in sucrose and corn syrup hard candies. However, they also observed that the same formulation did not readily release enough L-menthol during consumption. Complexation with β-cyclodextrin protected the L-menthol in the hard candy matrix but its release during consumption was unacceptably poor ( Reineccius *et al.,* 2004). A trade-off between flavor retention and release is therefore required while designing new flavor-added hard candy formulations.

Addition of multiple flavor compounds in hard candies is also possible. However, flavor release properties and flavor perception can be influenced by the interactions that may take place between the added flavor compounds. Schober & Peterson (2004) investigated the effects of delivery mode (single or mixture) of L-menthol and 1,8-cineole flavor compounds on release and perception properties of these flavors during consumption. Breath analysis and sensory time – intensity tests showed that separate addition of any of these compounds to hard candies provided more rapid release at a higher concentration than their addition as a mixture. Separate addition also increased the flavor intensity perceived by the consumer. Consequently, the mode of delivery affected the release kinetics of these volatile flavor compounds. This may be due to the variation in solubility of the two compounds thanks to the molecular interactions that occur between them during consumption when added as a mixture ( Schober & Peterson, 2004). In addition to interactions between flavor compounds, product inhomogeneity also effects flavor retention, release and perception. Here, two major factors, namely volatility of flavor compounds in the product base and mass transfer resistance between the product and air, control the release of flavor compounds from hard candy products ( De Roos, 2003). A homogenous distribution of volatile compounds within a hard candy matrix is therefore preferable. If the product is inhomogeneous, volatile compounds are compartmentalized which may cause problems. Some flavors are dissolved in vegetable oil and then incorporated into the candy matrix. In this way, volatility of the added compound is reduced, though the oil may induce a biphasic system resulting in an oily surface to the final product. In this case, volatilization of the flavor compound takes place directly from the oil phase rather than the whole product. Consequently, lower flavor retention could be observed in inhomogeneous hard candies. On the other hand, dissolution of flavor compounds in other substances such as propylene glycol may increase the volatility of the added flavor compound in its microenvironment whilst the opposite effect is observed in the hard candy matrix. The reason for this is the homogeneous mixing of propylene glycol in the hard candy matrix and thus, volatilization of the flavor compound occurs from the whole volume of the product rather than from a compartmentalized phase. Consequently, the total retention of that flavor compound in the candy matrix is increased ( De Roos, 2003). This information could be used to modulate the release rate of various compounds from a great variety of hard candy formulations.

### Recrystallization and stickiness during storage

Sugar recrystallization and/or increased stickiness are two main quality problems of hard candies encountered during storage. These undesirable changes in candy structures could be induced by temperature, RH or both ( Cardoso & De Abreu, 2004). Generally, storage conditions with high temperature and high RH promote graining or stickiness depending on the product formulation ( Nowakowski & Hartel, 2002). Hard candies are brought to glassy state during production in order to restrict molecular mobility that would induce textural deterioration during storage. However, changes in the external conditions (temperature, RH) may still initiate changes leading to quality deterioration ( Netramai *et al.,* 2018).

Doctoring agents such as corn syrups are added to hard candy formulations to retard sucrose crystallization during storage ( Hartel & Shastry, 1991). Glucose and fructose molecules introduced by corn syrup increase the hard candy mixture viscosity and inhibits sucrose crystallization. Corn syrup solids can also prevent the incorporation of crystallizing sucrose molecules into the crystal lattice ( Gabarra & Hartel, 1998; McFetridge *et al.,* 2004). Corn syrup sugars achieve this via impeding the diffusional motion of sucrose molecules and adsorbing onto the crystal surfaces. These sugars also decrease the solubility concentration of sucrose ( Tjuradi & Hartel, 1995) and the presence of glucose polymers in corn syrup contributes to the delay of sucrose crystallization onset ( Lees & Jackson, 1992). All these phenomena induced by doctoring agents extend the shelf life of hard candy products though doctoring agents including glucose and fructose are better humectants than sucrose. A sticky product due to excess water absorption during storage (lower T _g_) may thus be obtained at high glucose and fructose concentrations ( Iglesias *et al.,* 1997).

Temperature- and moisture-induced graining mechanisms result in subtle differences in the crystal structure. Graining induced by moisture uptake starts from the hard candy surface. A thin absorbed moisture layer forms at the surface and sucrose crystal nucleation starts in that layer. Water then starts to diffuse into the interior of the product and the grained portion increases ( Levenson & Hartel, 2005). On the other hand, temperature-induced graining typically starts internally when the storage temperature exceeds the T _g_ of the hard candy. Molecular mobility increases and crystals that were initially formed during production begin to grow in the candy matrix ( Liang *et al.,* 2007). Both cases are undesirable in terms of quality attributes and should be avoided by optimizing hard candy formulations, process parameters and controlling storage conditions.

**Figure 3.**
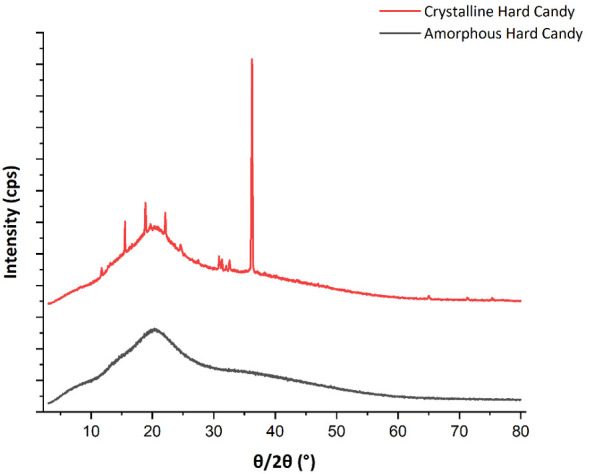
XRD profiles of hard candy samples with different crystallinity properties. The broad peak is from the amorphous component whilst the sharp peaks originate from crystalline regions.

Sugar recrystallization in hard candies can be monitored and analyzed using many analytical techniques. Microscopy is a widely used technique for detection and characterization of crystalline structures in food products ( Stanley *et al.,* 1998). Reflection and transmission simple light microscopy can be used for detection of crystals bigger than five µm however this technique requires separation of crystals from the product. For crystal size values between one and five µm, polarized light microscopy must be used to enhance the crystal detection ( Lans *et al.,* 2018). Additionally, confocal scanning laser microscopy is a more advanced technique capable of generating a three-dimensional view of the analyzed sample. In this technique, laser light illuminates the sample layer by layer. Confocal scanning laser microscopy could therefore be a better alternative for examination of crystal structures in hard candies. Detection and analysis of small crystals that are less than a few micrometers in size can be performed by electron microscopy such as SEM ( Bund & Hartel, 2010). Thermal techniques such as DSC can also be used for detection of crystallization in hard candies. Differences in temperature or heat flow associated with crystal formation or melting could be detected by DSC ( Roos & Karel, 1990). DSC is not however suitable for exact determination and differentiation of crystal types in food products. Another approach for detection and characterization of crystalline matter is spectroscopy. Spectroscopic measurements are nondestructive and require small sample volume. Typically applied spectroscopy techniques include X-ray diffraction (XRD), Raman micro-spectroscopy, nuclear magnetic resonance (NMR) and infra-red (IR) spectroscopy ( Bund & Hartel, 2010). These techniques can detect the presence of crystalline matter and differentiate the crystalline polymorphic structures. For instance, XRD produces spectra specific to each crystal type thus the ordering and packing of molecules in crystalline phases can be detected ( Labuza & Labuza, 2004). Differentiation of crystal polymorphic types is also commonly performed by XRD. Furthermore, XRD can be used to predict the degree of crystallinity within hard candy samples. Amorphous matter in the hard candies produces a characteristic hump with no sharp peaks due to random molecular orientations in the amorphous state. In contrast, distinct sharp XRD peaks are observed for crystalline material ( Roe & Labuza, 2005). XRD profiles of hard candies with different crystalline and amorphous phase contents are shown in Figure 3.

**Figure 4.**
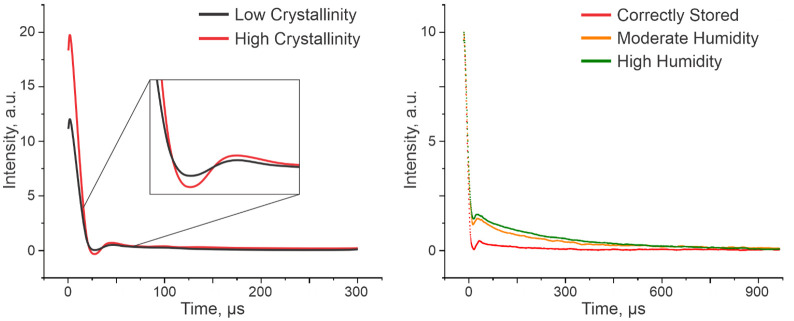
NMR Solid Echo signal presentation of hard candies. Left hand side is two different crystallinities with the insert showing a close up of the turning point. Right hand side shows hard candy which has been exposed in the short term to different levels of humidity. An additional long relaxation is seen as water is absorbed resulting in stickiness but minimal loss of crystallization has occurred.

NMR relaxometry is another approach to characterize crystalline matters. In this technique a radio frequency pulse is applied to a sample in a magnetic field to excite the spins of protons in the molecules to a higher energy state. After removal of radio frequency pulse, excited nuclei relax back to their original energy state by releasing a non-radiative energy which is received by the NMR instrument ( Hashemi *et al.,* 2010). In this way, liquid, amorphous and crystalline parts of a sample can be differentiated by NMR relaxometry. NMR is already an official method to calculate solid fat content of foodstuffs (SFC) ( Teles Dos Santos *et al.,* 2014). Generally, the so called free induction decay (FID) signal is measured for such systems ( Günther, 2013). However, this approach is not suitable for samples in the solid state ( Dejong & Hartel, 2016; Le Botlan *et al.,* 1998). Application of alternative sequences such as Solid Echo and Magic Sandwich Echo has are beginning to gain popularity ( Grunin *et al.,* 2019). Such sequences allow NMR relaxometry to be used to characterize the crystallinity properties and moisture content of hard candies. Figure 4 shows the signal from solid echo NMR relaxometry measurements of hard candies with changing crystallinity values and differing moisture contents through storage at suboptimal relative humidiy (RH).

**Figure 5.**
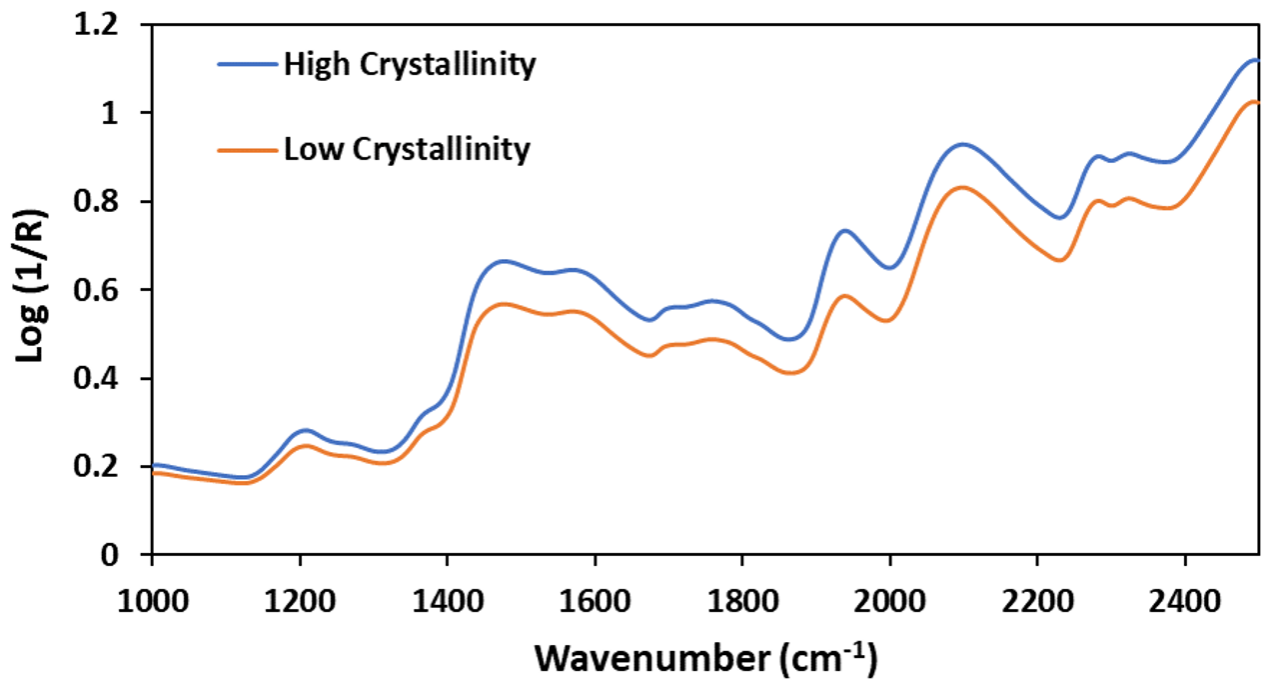
NIR spectra obtained from hard candy samples with different crystallinities.

Finally, Raman and IR spectroscopy techniques are suitable for obtaining information about crystals in food products ( Bund & Hartel, 2010). Figure 5 demonstrates typical near-infrared spectroscopy (NIR) spectra of hard candy samples with different crystallinities. In Raman spectroscopy for instance, a light of a specific wavelength excites the molecules to a higher vibrational energy state. The wavenumber shifts between the emitted and incident radiation are associated with distinct molecular vibrations specific to the molecules and their interactions with the environment. In this way, the spectra produced by this technique can be analyzed to detect and differentiate polymorphic structures of crystals ( Banwell & McCash, 1994).

## Future directions

Research on hard candies has generally focused on product formulation, process parameters and final quality attributes. There is however also a need for predictive measurements and models to optimize product formulations and industrial production processes. In this way, better process economics can be achieved for the industrial production of hard candies. Production of higher quality hard candies and improvement of the consumer acceptance are also possible with this approach. Recently researchers have prepared studies regarding novel and predictive approaches for hard candy formulation, production, packaging and storage. Some examples include optimization of hard candy formulations for longer shelf life by D-optimal mixture design ( Spanemberg *et al.,* 2019), application of image processing and convolutional neural networks (CNNs) for detection and classification of defective hard candies (color, shape and texture defects) ( Wang *et al.,* 2021) and application of mathematical models of moisture diffusion to estimate the shelf life of hard candies ( Spanemberg *et al.,* 2022). Such techniques are likely to contribute to the design of best possible hard candy formulations with desirable quality characteristics, longer shelf life, better process economics and consumer acceptance with reduced dependence on extensive traditional research and development. For instance, integration of computer-based image processing to industrial hard candy production lines may provide on-line, fast and nondestructive quality inspection instead of time consuming manual inspection performed by trained inspectors ( Wang *et al.,* 2021). The research in such fields however is still not at a sufficient level to see widespread adoption in factories at this stage. More studies focusing on these issues are therefore necessary to bring these new developments to the factory floor.

## Conclusion

Hard candies are a popular class of confectioneries with specific physicochemical, textural and sensory characteristics. Although hard candy manufacturing may seem like a straightforward process, production parameters should be carefully monitored and controlled to produce products with acceptable quality. Final product quality depends on many factors such as production method and parameters, initial composition of hard candy mixtures and addition of any colorants or flavors. Generally, high temperatures are required during production to reduce the final moisture content of hard candy products. Therefore, the types of added any colorant – flavor compounds should be resistant to high temperatures as much as possible. Furthermore, storage conditions have a great impact on the stability (shelf life) of the hard candies. Sucrose recrystallization and stickiness are the two main problems encountered during storage. Storage temperatures above T _g_ and high RH are responsible for graining and other undesirable textural and sensory alterations such cold flow, sticking and flavor loss. Doctoring agents such as corn syrup are generally added to hard candy formulations in order to reduce the risk of graining during storage, though the level of such doctoring agents should be carefully controlled, to prevent excess moisture absorption leading to stickiness during storage. In this review, production methods and parameters as well as quality parameters of hard candy products were addressed along with a discussion of the characterization techniques suitable to assess them.. There has been great development in the hard candy research as discussed in this review though new research in areas such as predictive modelling of hard candy formulations and use of image processing techniques to detect quality defects in hard candies are still needed. Further research in these fields would also contribute to the industrial production of hard candies in terms of process economics and consumer acceptance.

## Ethics and consent

Ethical approval and consent were not required.

## Data Availability

No data are associated with this article.
