# Supplementary material for: Hard Candy Production and Quality Parameters: A review
Source: Open Res Eur. 2024 Mar 26;4:60. [Version 1] doi: 10.12688/openreseurope.16792.1 (PMC11214042; doi:10.12688/openreseurope.16792.1)
Supplement: Peer review history

# Reviewer report: Reviewer response for version 1

**DOI:** 10.21956/openreseurope.18143.r39850

**Published:** 2024-06-28

**Authors:** Indri Juliyarsi

**Affiliations:** 1 Universitas Andalas:, Padang,, Sumatera Barat, Indonesia

This paper reviews the literature on the production and parameters of hard candy. While a comprehensive review is provided, additional references, especially those related to storage, are needed. In conclusion, a summary of the key findings is presented. However, it is recommended that future research include references regarding the storage of hard candy.

Is the review written in accessible language?

Yes

Are all factual statements correct and adequately supported by citations?

Yes

Are the conclusions drawn appropriate in the context of the current research literature?

Partly

Is the topic of the review discussed comprehensively in the context of the current literature?

Partly

Reviewer Expertise:

My research is focused on processing livestock products that are functional foods. These include milk processing, waste processing and food packaging handling.

I confirm that I have read this submission and believe that I have an appropriate level of expertise to confirm that it is of an acceptable scientific standard.

# Reviewer report: Reviewer response for version 1

**DOI:** 10.21956/openreseurope.18143.r41099

**Published:** 2024-06-17

**Authors:** Diana Lo

**Affiliations:** 1 Bina Nusantara University, Jakarta, Indonesia

All aspects has been written comprehensively. However there are two concerns I would like to raise:

 1. Citation of picture. The picture need the citation if it was obtained from other research paper. if it is based on author research, please explain more about the research, for example what type of hard candy used in the research

 2. Definition of hard candy. The definition of hard candy is "a product in glassy amorphous state below glass transition temperature (Tg )" However, Table 1 showed many of the product that may not belong to hard candy as it is not glassy. Please clarify the definition of hard candy or it is recommended to change the caption of Table 1.

Is the review written in accessible language?

Yes

Are all factual statements correct and adequately supported by citations?

Partly

Are the conclusions drawn appropriate in the context of the current research literature?

Yes

Is the topic of the review discussed comprehensively in the context of the current literature?

Yes

Reviewer Expertise:

Food Technology, sugar replacement

I confirm that I have read this submission and believe that I have an appropriate level of expertise to confirm that it is of an acceptable scientific standard.
